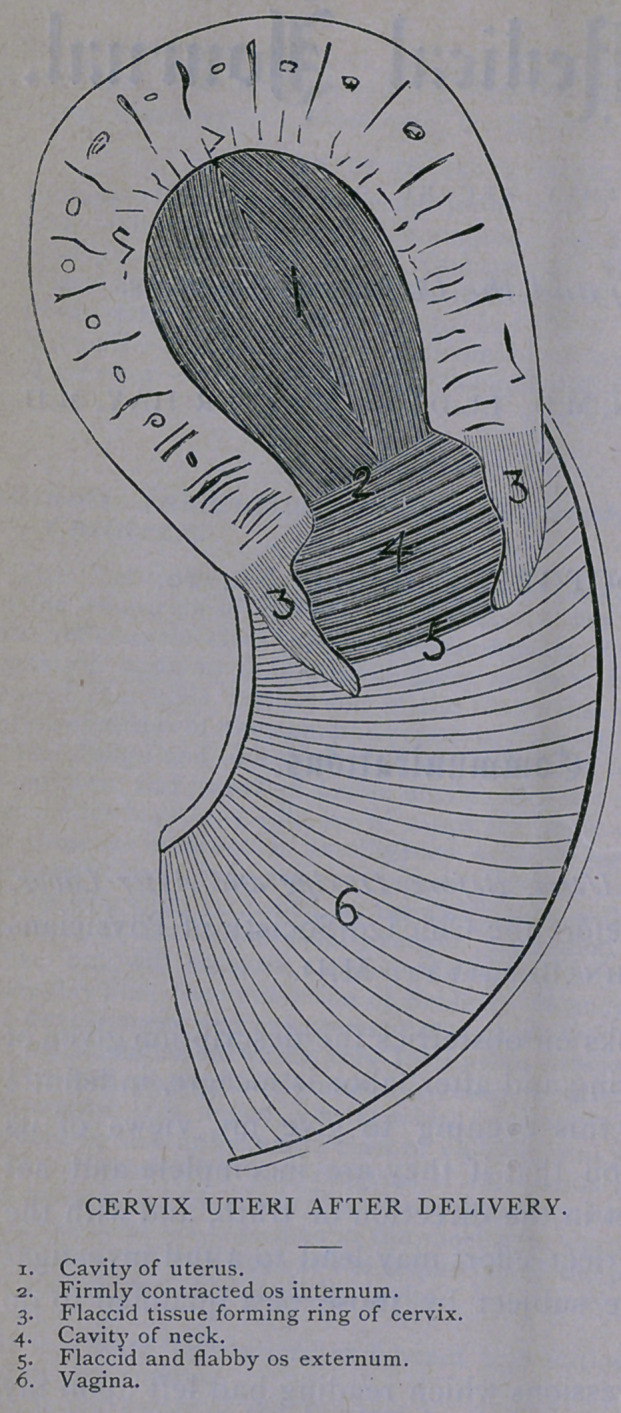# The Cervix Uteri, before, during and after Labor

**Published:** 1873-10

**Authors:** John Bartlett


A MONTHLY RECORD OF

*Medicine, Surgery and the Collateral Sciences.*

EDITED BY J. ADAMS ALLEN, M.D., LL.D.; AND WALTER HAY, M.D.

VOL. XXX. — OCTOBER, 1873. — NO. 10.

### Original Communications.

ARTICLE I. — *The Cervix Uteri, Before, During and After Labor.* Read July 14, 1873, before the Chicago Society of Physicians and Surgeons, by JOHN BARTLETT, M.D.

In many of the text books on obstetrics the description given of the cervix uteri before, during and after labor is meagre, indefinite or erroneous. I propose this evening to give my views of its relations, with the conviction that if they are incomplete and not free from error, they are yet in the direction of truth, and with the expectation that this imperfect effort may lead to a full investigation and elucidation of the subject by those best qualified to do it justice.

I will first give the impressions which reading had left upon my mind as the commonly received relations of the cervix; then state the views which observation has suggested to me as correct; and finally, point out the interest or importance which these may have for the obstetrician.

As to my early views, which, it will be inferred, I deem as fairly deducible from the statements to be found in the books: in pregnancy, I regarded the cervix as remaining undeveloped until

CERVIX UTERI AFTER DELIVERY.

1. Cavity of uterus.

2. Firmly contracted os internum.

3. Flaccid tissue forming ring of cervix.

4. Cavity of neck.

5. Flaccid and flabby os externum.

6. Vagina.

within two weeks of term, contributing nothing to the cavity of the gravid uterus. That at that time it began to expand from below upwards, gradually disappearing from the touch, till nothing remained of it but a cushiony circle, or, at most, a narrow, flattened, sphincter-like ring of tissue.

Observing dilatation of the os and retraction of the tissue forming it to be coincident and allied phenomena, I naturally associated with the complete dilatation of the os the entire disappearance of the neck; so that, at the instant of complete dilatation, in the language of Cazeaux, "the uterus and vagina formed but a single uninterrupted canal."

After labor, I thought that the contractions of the uterus, taking place equally in all parts going to make up its walls, formed it into a body the shape of which, exteriorly and interiorly, was not materially different from that of the distended condition. I believed that the margins of the os uteri which I had last felt still expanding and vanishing above the descending head, had kept pace in contraction with the body of the uterus, and that its firmly contracted, thick and rigid margin could be felt after labor immediately continuous with the upper end of the vagina. In time, according to my idea, as involution progressed, the uterine cavity reached by the finger beyond the os just referred to, gradually contracted, and became divided into body and neck, as before labor; the os described meanwhile contracting, and assuming substantially its original shape and relations below the vaginal attachment.

These views I now regard as entirely erroneous. For them, observation has substituted these which follow:

In regard to the cervix before labor, I look upon all that part of the uterine walls projecting in vertex cases, like an inverted dome, into the vagina, and which may be felt enclosing the head, as the largely developed vaginal portion of the neck; that is, I regard all that uterine tissue lying below the attachment of the vagina, and which may be traced by the finger moving in any direction from the os uteri toward the vaginal circumference, as the vaginal portion of the cervix.

These statements, it must be noticed, have reference to the vaginal portion of the cervix only. There is reason to suppose that the uterine two-thirds of the neck are developed in like proportion. The extent of this development may be in a measure estimated by conditions or events accompanying natural and unnatural states of the womb after delivery.

I regard the vaginal portion of the cervix, at the time of the passage of the fœtus, as a collar of appreciable depth not in the same plane with the vagina, but projecting into it.

After the birth of the child, this fleshy band does not contract and form with its free margin the rigidly contracted orifice discovered after natural labor and generally somewhat indefinitely called the "os." Its tissue differs from that of the uterus, its power of contraction is much less. As a result, after delivery it may be felt as a flabby, floating collar hanging from the uterus into the vagina, "like a section of large intestine." Continuous with this fleshy cylinder, externally and above, is the attachment of the vagina; continuous with it, above, and within, is the rigidly contracted os internum. The involution of the post partum cervix soon begins, and at the expiration of ten or twelve days it will have assumed its ordinary form and relations.

As it was from examinations per vaginam, after labor, that this state of the cervix was made known to me, and as by this means inquirers will seek to verify these statements, it may be well to make a detailed reference to such an exploration. If, after delivery, the uterus being contracted, a finger be introduced into the vagina, it may pass directly up to the "os," without coming in contact with any structures contravening the idea of the simple relation of the parts at first thought by me to be correct. If an examination be made critically, it will be recognized that the finger encounters an illy-defined orifice or slit, the margins of which are soft, flaccid and easily movable; penetrating these folds, the os is felt higher up than the normal position of the os externum by about the length of the floating cervix. The end of the finger resting upon this contracted ring, the os internum, the edges of which project prominently and abruptly inward, is not of course in the vagina, it is in the canal of the cervix. If it be moved outwardly from the pelvic axis in any direction, it will encounter a loose tissue which may at first be supposed to be the walls of the vagina; but if this surface be followed downward for an inch or an inch and a half, a free margin will be reached, which, upon being traced, will be found to be the rim of a fleshy ring. The surface encountered is the inner wall of the canal of the vaginal portion of the cervix; the margin traced is the still widely expanded, imperfectly contracting os externum.

The dimensions of the vaginal portion of the cervix are variable. A radius or meridian of the ante partum cervix, measured from the os to its circumference, is generally not less than two inches, and occasionally, when the mouth of the womb is far removed from the pelvic axis, the larger radii would measure five inches or even more. During the passage of the child, the length of the neck, usually about three-quarters of an inch, in some abnormal conditions is so great that its margins may be protruded at the vulva. After delivery, the infra vaginal cervix, commonly an inch, or an inch and a half, in length, in cases of extreme hypertrophy of the neck, has been found longer than the vagina.

The facts here detailed have a bearing upon several points of obstetrical theory and practice. The different action and condition of the body and neck of the uterus here pointed out may be considered another striking evidence of the correctness of the distinctions which physiologists and practitioners have drawn between these portions of the womb.

The views here given are important as bearing upon the long disputed question of the mode of development of the cervix in pregnancy. On this point, obstetrical writers seem to be divided between two opinions; one set maintaining that the neck develops in expanding from above downward, and becomes part of the uterine cavity as early as the sixth month. Others, as Stoltz, Cazeaux, and Chailly, pronounce this view an error, and declare that the neck forms no part of the uterine cavity till within two weeks of term, when it expands from below upwards, disappearing gradually, and at the end remaining only as a cushiony circle.

In the light of the views here expressed, the second of these theories, which I believe is now most favorably regarded, is altogether erroneous; and the first fails to point out the whole truth. I think that early in pregnancy the cervix begins to develop throughout its entire extent; that gradually the upper portion of it expands and becomes a part of the uterine wall. Meanwhile, (and this is the fact which I think is entirely overlooked by Stoltz and Cazeaux), that portion of the neck which remains unexpanded has grown, as it were, on account of its *preparatory* development, to the length of the original cervix; finally, the whole is encroached upon and taken up to form the uterine walls. To put the idea in different language: early in pregnancy the neck is called upon to supply its quota to the enlarging body. Speaking somewhat figuratively, as ring after ring of tissue is demanded from the upper part of the cervix, the preparatory development in the remaining portion is such that the length of the neck is not apparently impaired, so that, what remains of it as late as two weeks before labor has been mistaken for the entire infra and supra vaginal cervix, whilst the loss by the continual transfer from the upper portions of the neck to the uterine walls has entirely escaped notice. That circle of the neck which corresponds at the time of an examination to the limits of its expansion, is regarded by writers as the os internum. The os internum is of course, as before labor, above the attachment of the vagina, and, near term, far removed from the examining finger. The apparent constriction taken for it, is simply that point in the cervical walls, marking the constantly descending line of demarkation between the expanded and yet unexpanded portions of the neck.

In my early examinations the contracted post partum os was mistaken for the os which I had felt before labor, the os externum; and all that structure, consisting, at the beginning of labor, of the vaginal portion of the cervix, measuring several inches in depth, and forming the os which I had so patiently watched a short time before, was entirely overlooked and lost sight of, and by a species of jugglery as it were, a second os, which I knew nothing of, was imposed upon me for the one with which I had become from long watching familiar.

It is important, in lacerations of the os uteri, to know the site of the bleeding surface. In some cases of hemorrhage, occurring in the earlier years of my practice, which by a process of exclusion were referred to a laceration of the neck, I had no other idea than that the oozing proceeded from the little irregularities in the contracted os internum, when in all probability there were surfaces bleeding, such as I have since recognized under like circumstances, in the torn cervical rim, as large as the lower half of the little finger. In cases where the hemorrhage is referred to such lacerations, the practitioner will do well to trace the margin of the os externum; if it be much torn, the rent cannot escape detection, and the application of styptics may readily be made.

Some authors refer irregularities of outline of the os tincæ in multiparæ to lacerations of the mucous membrane of the os during labor. Is it not much more probable that such irregularities are dependent upon more decided lesions of the neck? My experience would indicate that lacerations are more frequent than is commonly supposed. The cases of hemorrhage proceeding from these injuries of the cervix which have fallen under my notice, have yielded to remedies intended to secure contraction of the uterus. As the mass of the vessels supplying this part of the womb pass through the tissue of the os internum, it is manifest that the want of contractility of the neck is in a measure compensated for by the mechanical constringing of its vessels by the contraction of its upper orifice.

In certain forms of rigidity of the cervix the relations here given would suggest an early resort to the knife. In those cases in which labor is delayed by the os girding the neck of the child, in all probability, as suggested by some writers, the constricting orifice is the os internum. In this connection, this inquiry has occurred to me: Does mechanical irritation of the os externum tend to excite contraction of the os internum?

The idea here presented of the mode of development of the cervix is of interest in relation to placenta prævia. By some, doubtless, it may be objected that the presence of the placenta over the os uteri is *prima facie* evidence that my opinions as to the development of the neck are incorrect. To my mind, there remains but the acceptance of the palpable facts, and the recognition in them of another striking example of the extraordinary power which nature has to accommodate herself to circumstances. The imperfectly contractile character of the tissue of the cervix deserves more attention than it has yet received in considering the growth upon it of the placenta; and it is possible that some fact of practical value in regard to the management of placenta prævia may be adduced from an improved knowledge of the nature of the tissue to which it is attached. By even the most recent writers, as Barnes and Thomas, it is distinctly stated that the os internum is below the placenta prævia; according to my views, the placenta is below the zone of fibres developed from and representing the os internum. In accordance with the theory of cervical development here stated, the attachment of the placenta to the cervical zone is the more recent, and, inferentially, the less firm in a line and direction *from* the os *toward* the placental margins, a circumstance indirectly favoring the suggestion of Barnes to detach the placenta from the zone of the neck in certain cases. Consideration of the statement above made in regard to the contraction of the fibres of the os internum as a cause of arrest of hemorrhage from the lacerated cervix, may confirm and elucidate the proposition of Barnes, "that the one constant condition of physiological arrest of flooding [in placenta prævia] is contraction, active and tonic, of the muscular structure of the uterus."

There is another condition upon which the facts here presented have a direct bearing. I refer to hour-glass contraction of the uterus. Before proceeding to any remarks on this subject, I will call your attention to certain considerations in regard to the position of the os internum. In the contracted state of the womb as stated, we find it in deep reach of the finger, and but little above the attachment of the vagina. But it must be remembered, that the relative altitude above the os externum of those fibres of the walls of the expanded uterus, the contraction of which forms the internal os, is not ascertained, and that it must vary with the degree of expansion or contraction of the womb. Thus, in a flaccid condition of the organ after delivery, it may be several inches above the vaginal junction. The statements of writers as to the seat of hour-glass contraction, and the part of the uterus by which it is caused, are discrepant and confusing. Much of this misunderstanding and obscurity, it is believed, may be cleared away in the light of facts contained in this paper. From a person proposing to teach the true nature of this abnormal condition of the uterus, an answer to these questions should be expected: What was the condition in your cases of the os externum? Of the cervix uteri? Of the os internum?. What was the probable altitude of the os internum above the os externum? And where, relatively to the internum, was the stricture?

I may state in this connection that, as the womb is capable of becoming flaccid as late as two weeks after labor, the os internum may be expected to be liable to corresponding changes after a like interval.

I desire, before closing this essay, to make some suggestions intended to account for the want of more general recognition of the correct anatomy of the cervix after labor. Its true condition has escaped the observation of many, because there is not very often occasion to make examinations which would disclose it. For the most part, after delivery, the fingers are introduced to ascertain if the patient be well cleared, and if such be the case, no further examination is made, or, in the event of the exploration of the vagina being again necessary, it is generally rendered so by an accident unfavorable to critical exploration. Again, it must be remembered that the condition here pointed out is dependent upon, and not to be recognized without a contraction of certain parts of the womb.

I have been asked, why has not the peculiar anatomy which you describe as distinguishing the post partum cervix been observed by the pathologist? I repeat, that the distinction indicated is dependent upon a vital contractility; when that passes away, the distinction ceases.

A few words more. It will doubtless be inferred that I regard these views as novel. Whilst it is manifest that the true condition of the cervix uteri during and after labor is practically familiar to many standard writers, so far as my knowledge of the literature of the subject goes, no one has connectedly set forth the facts here stated.

I am well aware, however, that my means of reference are too limited to justify me in saying that they are not well known. Of this only am I assured, that however often these ideas may have been taught, a lesson from them is not now amiss.

## Figures and Tables

**Figure f1:**